# Supplementary material for: Are carbon emissions trading and green financial instruments synergistic? -Comprehensive quantitative research based on content analysis
Source: PLoS One. 2024 Mar 7;19(3):e0298601. doi: 10.1371/journal.pone.0298601 (PMC10919601; doi:10.1371/journal.pone.0298601)
Supplement: S1 Data — (ZIP) [file pone.0298601.s001.zip › Data/地级市Z_F工作报告.zip!/║┌┴·╜¡/╣■╢√▒⌡╩╨/└┤╫╘╣1⁄2╓┌║┼í╛┬φ┐╦╩2╛▌═°í┐_2009.docx]

各位代表：

　　我代表市人民政府，向大会作工作报告，请予审议。并请政协委员和列席会议的同志提出意见。

　　2008年工作回顾与总结

　　过去一年，是哈尔滨市经济社会发展进程中不平凡的一年。全市上下深入贯彻落实科学发展观，按照省委提出的“加快发展、当好龙头”的总体要求，紧紧围绕建设“三个适宜”现代文明城市目标，牢牢把握发展机遇，积极应对各种挑战，千方百计加快经济社会发展，全心全意解决民生问题，圆满完成市十三届人大二次会议确定的各项任务。

　　———全面贯彻落实国家宏观政策，着力保持发展的协调性和稳定性，国民经济持续快速增长。预计（下同）全年实现地区生产总值2800亿元，比上年增长13.2%，连续19个季度保持13%以上的增幅。财政一般预算收入280.5亿元，按可比口径增长27%，其中地方财政一般预算收入164亿元，按可比口径增长27.2%。全社会固定资产投资1340亿元，增长30%。社会消费品零售总额1258.7亿元，增长21.5%。

　　———大力推进结构调整,加快产业升级步伐，三次产业协调发展。三次产业结构优化，由上年的14.3∶37∶48.7调整为13.9∶36.8∶49.3。农业生产实现新突破。完成农林牧渔业总产值670亿元，增长7%。战胜严重春旱，粮食生产再创历史新高，总产量达到210.8亿斤。畜牧业实现产值310亿元，增长6.5%，占农林牧渔业总产值的比重由上年的45.6%提高到46.3%。农业产业化经营机制不断完善，带动作用明显增强，规模以上龙头企业由140户增加到157户。工业经济迈上新台阶。规模以上工业实现增加值500亿元，增长15%，高于地区生产总值增幅1.8个百分点。装备制造、医药、食品和石化4个优势产业增加值占规模以上工业比重达到83%。第三产业取得新发展。实现增加值1380亿元,增长13.9%。接待国内外旅游人数3019.7万人，增长19.9%；实现旅游总收入245.9亿元，增长20.3%；哈尔滨市被评为“中国十大节庆城市”和“2008年度风情城市”。会展业发展势头强劲，成功举办“哈洽会”等一批大型展会。龙运物流园区、新透笼购物广场、南极国际商贸城等一批物流项目开工建设。金融业发展步伐加快，韩国国民银行哈尔滨分行落户哈尔滨市，兴业银行哈尔滨分行、中信银行哈尔滨分行挂牌营业，哈尔滨银行在天津等地设立了分行，村镇银行试点在巴彦县启动，天一药业、中强科技在美国证券柜台交易市场升为证券市场，泰富电器在纳斯达克增发成功，人和商业控股成功上市。截止2008年11月末，全市存款余额4108.6亿元，比上年同期增长23.2%；贷款余额2603.5亿元，增长7.4%。非公有制经济发展实现新跨越，增加值占地区生产总值比重首次超过50%。

　　———扩大改革开放，外借助力内增动力，经济活力持续增强。国有企业改革扎实推进，一些重点企业战略性重组取得实质性进展。厂办大集体改革全面启动，完成了具备条件的 660户企业改革范围界定审批。筹资14.96亿元，全面完成解欠工资任务。积极争取国家和省支持，获各类专项资金68.9亿元。招商引资取得新成效。新批外商投资企业82户，实际利用外资5.43亿美元，增长22.3%；国内经济合作到位资金294.9亿元，增长40.3%。对外贸易增长较快。实现进出口总额35.8亿美元，增长20%，其中出口18.5亿美元，增长12%。科技创新步伐加快。42项自主知识产权项目被国家批准为重点新产品，连续两年居副省级城市首位；12项科技成果获国家科学技术奖励。高新技术产业产值1350亿元，增长25%。科技进步对经济增长的贡献率由上年的46%提高到49%。

　　———努力扩大投资，增强发展后劲，区域经济增长动力依然强劲。100个市重点项目完成投资260.6亿元，投资完成率高于上年28.5个百分点，投资额和增速达到历史最好水平。新增规模以上工业企业227户，是前4年的总和；地方工业实现增加值292亿元，增长21%，高于全市规模以上工业增幅6个百分点。哈开发区实现工业总产值1150亿元，增长20.9%。哈大齐工业走廊哈尔滨段39个投资亿元以上项目全部开工建设，呼兰河新型工业园区被确定为核心示范园区。群力、哈西、松北等新区开发建设取得积极进展。

　　———强化规划建设管理，打造干净有序城市，城乡载体功能和环境质量得到提升。编制完成《印象·哈尔滨规划》、《城市公共服务设施规划》、《沿江产业带规划纲要》等14类31项规划，江南城区实现控详规划全覆盖。重大基础设施建设取得新突破。轨道交通一期工程开工建设，松浦大桥全面开工，三环路哈阿立交桥主线桥建设、太平大街等路网升级达标改造、防洪纪念塔东广场改造工程全面完成。市政公用设施建设取得新进展。磨盘山供水二期、城市气源转换和堤防升级达标、既有建筑立面装饰节能墙体改造等工程有序推进；江南城区饮用磨盘山水居民用户达到62%，40万居民用户用上天然气，城区集中供热普及率达到64%。“干净有序城市”建设扎实推进。8类31个方面迎 “大冬会”城市环境整治任务全面完成，背街巷道、城乡接合部、铁路沿线、江河两岸环境卫生状况有所改善；新增绿地1065.2公顷，新植树木127.4万株，改造居民庭院556个，新建市民休闲健身广场56个；调整、延伸公交线路19条，更换公交车辆468台；道路“畅通工程”进展顺利，数字城管网格化管理体系和快速反应机制不断完善。农村基础设施建设稳步推进。开工建设高等级路面农村公路3213公里，通乡通村公路硬化率分别达到97%和77%；新建乡镇客运站14个，开辟、调整、延伸农村客运班线14条，改造农村危桥115座，7万农村群众出行难问题得到解决；建设农村饮水安全工程173处。新打抗旱井3.2万眼，维修、新建蓄水工程120处。

　　———实施可持续发展战略，强化节能减排和环境保护，生态环境持续改善。松花江流域水污染防治规划项目全面展开，24个国家规划治污项目全部开工，建成文昌三期、阿城等6个污水集中处理厂，启动建设群力污水处理厂，重点工业企业和医院废水排放全部达标，14条一级支流水质达标率提高14个百分点，松花江出境水主要污染指标降低2%。城市空气环境优良天数达到308天。节能减排取得新成效。完成投资千万元以上节能项目6个，淘汰10户企业落后产能，完成万元生产总值综合能耗下降指标。实施减排项目33个，水、气主要污染物削减量分别超额完成年计划的10%和15%。全面完成三北防护林等7项工程，造林13.2万亩，超年计划32%。治理水土流失65万亩。建成省级以上生态示范区5个，省级环境优美乡镇4个、生态文明示范村18个。

　　———着力改善民生，促进社会和谐，人民生活水平稳步提高。60项惠民行动项目如期完成。教育事业稳步推进。全部免除城乡义务教育阶段杂费。重点扶持农村教育，新建10所农村寄宿制学校。普及九年制义务教育形成的3.5亿元债务全部化解。职业技术教育发展较快，办学水平明显提高。城乡困难家庭和进城务工人员子女平等接受义务教育。新建、改扩建45个社区卫生服务机构和73个乡镇卫生院，城乡医疗卫生服务能力有新提高。县域城镇居民基本医疗保险试点全面启动，新型农村合作医疗基本实现全覆盖，参合率达到96%。农村药品供应网站建设实现全覆盖。低生育水平持续稳定，人口自然增长率控制在4.5‰。新建、改建91处社区公益用房，社区办公、活动条件得到改善。新增城镇就业11万人，转移农村劳动力116.2万人。零就业家庭成员托底安置动态为零。城镇登记失业率预计控制在3%以内。社会保障体系不断完善，基本养老、失业、医疗、工伤、生育保险覆盖面进一步扩大；投入1.5亿元，救助困难群众88万人次;城乡低保标准得到提高。新建农村敬老服务中心23所，市区全面推行居家养老模式。实施“好民居工程”，加快棚户区改造，采取“预约式拆迁”、“股转租”等多种模式，完成棚户区拆迁面积237万平方米。拆除农村泥草房4.39万户，新建砖瓦房4.09万户。解决廉租住房5355户，是前五年的1.61倍，城市人均住房使用面积8平方米以下的廉租户住房实现应保尽保。采取政府组织建设和货币补贴相结合方式，为3195户家庭提供经济适用住房。城市居民人均可支配收入增长14.2%；农民人均纯收入增长17.6%。

　　———弘扬先进文化，强化公民思想道德建设，“文明城市”建设取得成效。深入开展社会主义荣辱观教育，和谐基础创建活动成效显著。“拥抱大冬盛会，奉献火红青春”、“文明礼貌在冰城”等系列活动蓬勃开展。成功举办第29届哈尔滨之夏音乐会系列活动和“改革开放30周年”大型电视文艺晚会；承办第8届全国声乐比赛，并使这项赛事落户哈尔滨；市属专业剧院(团)获得13项国家级、51项省级奖项。36项民俗文化入选第二批市级非物质文化遗产名录。文化体制改革有新进展。新建社区图书馆16个，完成7个区文化馆、12个乡镇综合文化站、10个村综合文化室配套建设。架设有线电视光缆500公里，覆盖郊区20万用户。双拥工作再创佳绩，第六次荣获“全国双拥模范城”称号。

　　北京奥运会火炬在哈尔滨市安全传递。高质量完成赛事场馆设施建设等迎接“大冬会”各项筹备工作。结合“大冬会”开幕倒计时一周年、100天，开展了“迎奥运、迎大冬、促发展” 系列文体活动，提升了市民文明素质，营造了浓厚的迎赛会氛围。安装社区体育健身路径1400件（套），带动了全民健身运动的全面开展。竞技体育运动水平提高，在全国第11届冬运会上，哈尔滨市取得金牌、奖牌总数和团体总分3个第一。在北京奥运会上，哈尔滨市运动员焦刘洋夺取女子200米蝶泳银牌并打破世界纪录。

　　四川汶川特大地震发生后，全市人民情系灾区、踊跃捐助，累计为灾区募集款物折合人民币2.72亿元。及时派出740名公安特警、消防官兵和36名医疗专家、19名建筑地震专家奔赴抗震救灾第一线，抢救受灾群众1020人、救助病患3400多人。市公安局特警支队荣获“全国抗震救灾英雄集体”称号。

　　———坚持依法治市，推进依法行政，民主法制建设得到加强。自觉接受人大依法监督和政协民主监督，人大代表建议、政协委员提案办复率100%。全年提请市人大常委会审议地方性法规草案6件，制定政府规章32件。全面开展行政执法依据梳理和行政权力清理工作。强化行政执法监察和效能监察，政府绩效评估体系初步建立。推行行政执法责任制，规范了行政权力运行。深入实施《哈尔滨市行政机关领导问责暂行办法》，勤政廉政建设取得新成效。加强电子政务建设，推进政务公开，政府工作透明度和公共信息服务水平得到提高。“平安哈尔滨”创建活动取得成效，各类犯罪案件发案总量下降3.8%。开展“安全隐患治理年”和“安全隐患排查整治”专项行动，强力推行查处违法违规行为“三十个一律”措施，安全事故总量下降51.6%，死亡人数下降13%。创新行政复议受理方式，建立人民建议征集绿色通道，畅通群众诉求渠道。坚持主要领导包案和信访接待日制度，积极妥善处理各类社会矛盾，确保了重要敏感时期全市政治安定和社会稳定。“12345”市长公开电话开通运行。

　　妇女儿童、新闻出版、民族宗教、人事、统计、审计、外事、侨务、对台、老龄、气象、地震、档案、人防、民兵和预备役、地方志、残疾人等项工作也取得长足进步。

　　各位代表，过去一年成绩的取得，是省委、省政府正确领导、关心帮助的结果，是市委统揽全局、科学决策的结果，是市人大依法监督、鼎力支持和市政协民主监督、参政议政的结果，是全体市民同心同德、开拓创新的结果，是中省直驻哈企事业单位以及社会各界积极配合、大力帮助的结果。在此，我代表市人民政府，向全市广大工人、农民、知识分子和干部，向全体纳税人，向各民主党派、工商联、无党派人士和社会团体，向驻哈人民解放军、武警官兵和公安干警，向所有关心、支持哈尔滨发展的同志们、朋友们和海内外友人，表示崇高的敬意和衷心的感谢！

　　各位代表，过去的一年，虽然哈尔滨市经济社会发展取得了一些成绩，但我们清醒地认识到，按照科学发展观的要求，与发达地区相比，用广大人民群众的殷切期盼衡量，还存在一些亟待解决的突出矛盾和问题。主要是：长期形成的体制性、机制性和结构性等深层次矛盾和问题尚未得到根本解决；受国际金融危机影响，工业经济增速放缓、效益下滑，扩大出口、利用外资难度加大；融资渠道不畅，融资规模增长缓慢，一些重大项目建设资金短缺矛盾亟待解决；劳动力供求矛盾加剧，就业再就业形势严峻；保持农业稳定发展、农民持续增收难度加大；公共财力较弱,公共产品和服务供给不足；一些政府部门职能转变滞后，有些政府工作人员公仆意识、责任意识、服务意识不强，工作作风不实、效率不高，不作为、乱作为、中梗阻问题仍很突出；社会不和谐因素依然存在，维护社会安全稳定的任务还很艰巨。对此，我们要高度重视，努力在今后工作中认真研究解决。

　　2009年面临的形势和任务

　　今年在哈尔滨市发展史上将是不寻常的一年。作为全国解放最早的大城市，做好纪念国庆60周年系列活动对哈尔滨市具有特殊重要意义；作为第24届世界大学生冬季运动会承办城市，哈尔滨市将成为全世界瞩目的地区之一。

　　今年也将是既富于挑战又充满机遇的一年。国际金融危机尚未见底；我国经济增速下滑已经成为当前经济运行中的主要矛盾，今年可能是进入新世纪以来哈尔滨市经济发展最困难的一年。尽管面临严峻的宏观环境，但也存在一些有利因素。从外部支持看，国家实施“保增长、扩内需、调结构”战略，实行积极的财政政策和适度宽松的货币政策，为哈尔滨市扩大投资、加快发展创造了更为有利的宏观环境；党的十七届三中全会出台一系列惠农强农政策，省实施“千亿斤粮食产能工程”，将促进哈尔滨市农业和农村经济提速增效。从自身条件看，哈尔滨市经济自改革开放以来持续快速发展，特别是近15年连续保持两位数增长，为今年经济稳定运行奠定了坚实基础；哈尔滨市地域辽阔，城乡二元结构明显，城市化、工业化、市场化需求强劲，发展潜力很大；哈尔滨市科技实力较强，可以使我们在新一轮产业调整中抢先布局，提高自主创新能力，并积极转化为现实生产力；哈尔滨市实施追赶战略初见成效，去年前三季度，在全国15个副省级城市中，哈尔滨市有10项主要经济指标位次前移，有的指标呈明显上升态势。这些都为哈尔滨市经济平稳较快增长、又好又快发展奠定了坚实基础。只要我们进一步增强机遇意识，审时度势，沉着应对，把措施谋划得更周密一些，把工作做得更扎实一些，就一定能够把哈尔滨的改革发展不断推向前进。

　　2009年工作总体要求是：以邓小平理论和“三个代表”重要思想为指导，牢固树立和落实科学发展观，全面贯彻党的十七大、十七届三中全会和中央、省委、市委经济工作会议精神，紧紧围绕保增长目标，解放思想、“危”中求“机”、趋利避害、逆势前行，充分依托国家实施扩大内需战略，进一步加快转变发展方式、优化经济结构，进一步推进项目建设、扩大投资规模，进一步深化各项改革、提高开放水平，进一步加快以改善民生为重点的社会事业发展，确保经济平稳较快增长，努力推动经济社会又好又快发展。

　　综合考虑各方面因素，今年全市经济社会发展的主要预期目标是：地区生产总值增长12.5%，力争增长13.5%；规模以上工业增加值增长14%，力争增长15%；地方财政一般预算收入按可比口径增长16%左右；全社会固定资产投资增长35%左右；社会消费品零售总额增长15%左右；居民消费价格指数控制在104左右；市属进出口总额增长15%左右；实际直接利用外资额增长10%左右；国内经济合作资金实际到位额增长20%左右；城市居民人均可支配收入增长7%左右；农民人均纯收入增长7%左右；城镇登记失业率控制在4.4%以内；人口自然增长率控制在4.5‰以内；万元生产总值综合能耗降低5%；水、气主要污染物削减量累计完成“十一五”总削减量的70%。

　　围绕上述总体要求和目标，着力推进九项重点工作。

　　一、巩固和加强农业基础地位，努力实现农业稳定发展、农民持续增收

　　围绕稳粮、增收、强基础、重民生，进一步落实惠农政策，增强科技支撑，加大投入力度，优化产业结构，推进农业农村经济发展。

　　突出抓好粮食生产和农产品供给。保证粮食播种面积2580万亩，新增15万亩。启动实施增产50亿斤粮食产能工程，新增粮食产量8亿斤。新建国家标准粮田22万亩。抓好625万亩粮食高产创建工程，改造中低产田26万亩。加快建设水稻、玉米、大豆等10大优势产业带，新增无公害、绿色和有机食品认定面积200万亩，新增认证产品100个，建设绿色食品原料标准化生产基地8个、面积500万亩，良种覆盖率达到99%。加快发展“一村一品、一乡一业”特色农业。支持郊区发展园艺产业、都市观光农业和设施农业。加快畜牧良种繁育体系建设，提高规模化饲养水平，强化畜牧业质量安全体系建设，从源头上把好肉、蛋、奶等食品质量关。强化新型农业社会化服务体系建设，力争三年内建立健全乡镇或区域性农业技术推广、动植物疫病防控、农产品质量监管等公共服务机构。

　　强化农业基础设施建设。加快农田水利设施建设，夯实粮食增产基础。加快龙凤山等5大灌区续建配套、节水改造工程和沿江5个灌区整合工程，推进30座病险水库除险加固工程。继续落实对农民购置大型农机具和深松整地的补贴政策，提高农机装备水平，力争三年内实现水田全程机械化作业。继续实施科技入户和“三电合一”工程，全力抓好20个现代农业科技示范园区和20个农业信息化示范村建设。加强农业气象灾害监测预警，提高防灾减灾能力。

　　推进农业产业化经营。进一步做大做强农业产业化龙头企业，市级以上产业化龙头企业由54家发展到98家，增强产业化对农业现代化的拉动作用。鼓励和支持龙头企业与农民建立紧密型利益联结机制。积极引导工商资本、民间资本、外资投向农业和农村。加快推进绿色食品产业集聚区建设，做大拉长乳品、生猪、肉牛等10大产业链，继续打造一批国内绿色食品知名品牌。扎实推进有机米、菜、奶、肉四大基地试点，提高规模化、标准化、集约化水平。提高农民组织化程度，农民专业合作社发展到376个。

　　加快城乡一体化进程和农村各项改革。以打造全省城乡一体化发展先行区为目标，以城乡规划、产业布局、基础设施建设、公共服务一体化为重点，制定城乡一体化发展配套政策，推进“场县共建”、“场乡共建”、“林乡共建”试点，在郊区率先启动城乡一体化试点。继续抓好40个区域中心镇建设。启动和推进第二批新农村建设100个村试点。实施106个村扶贫开发整村推进。稳定和完善农村基本经营制度，抓好承包地确权、登记和发证工作。继续推进林权制度改革。探索构建农村土地流转交易平台，推进农村土地股份合作社试点，促进农村土地规模化、集约化和效益最大化经营。启动小额贷款公司试点，推进村镇银行建设，构建资本支农新平台。抓好郊区村庄资源整合、建设农民集中区试点，发展农村新型社区。积极化解乡村债务。扩大农村基层民主，加强基层政权建设。

　　二、坚定不移地走新型工业化道路，努力保持工业经济平稳较快增长

　　加快推进老工业基地振兴战略，充分发挥比较优势，进一步挖掘增长潜力，采取积极有效的防风险、保增长、调结构措施，做大做强工业经济。

　　加快产业结构优化升级。继续壮大装备制造、食品、医药、石化等优势产业，加快发展电子信息、生物医药和新材料、新能源等新兴产业，积极构建电站成套设备、汽车、飞机制造、专用设备、风电设备、铝镁合金新材料等24条产业链。深入实施大项目牵动战略，重点抓好100个投资亿元以上项目建设，启动中煤龙化、大飞机复合材料中心、哈飞新型乘用车等新建项目；推进东轻超大规格铝板带材、大成集团玉米深加工、电站集团核电装备、哈飞Z15直升机等在建项目建设；争取东安三菱自动变速箱一期、亚泰哈水一期、英联马利酵母及浸膏等项目竣工投产。工业固定资产投资增长25%以上。

　　加大力度调整空间布局。按照产业布局导向，推进哈烟厂、轻型车厂等企业搬迁改造。充分发挥国家级开发区的集聚带动作用，进一步整合资源，引导产业有序集中，打造经济增长新优势。大力整合区、县（市）工业园区，依托资源优势，着力打造特色产业园。推进哈大齐工业走廊哈尔滨段重点园区快速发展，突出抓好呼兰河新型工业示范园区、航空及汽车产业城、电子信息产业园、松北现代农机装备产业园和风电装备产业基地开发建设。

　　全力抓好工业生产。密切关注金融危机对工业经济的影响，继续完善预警预报监控体系，启动工业投资月报制度。健全重点企业联系机制，实施分类指导。支持市场需求旺盛的企业开足马力加快生产，增产增收。积极引导亏损企业加强管理，减本降耗，最大限度减亏增效。全力扶持困难企业保持生产并降低关停率。发挥新型工业化发展资金导向作用，支持优势企业加快技术改造，提高竞争力。进一步支持中小企业发展，全年新增规模以上企业100户。

　　强化企业信息化建设。深入实施企业信息化“百千万”工程，力争启动100户企业试点。加快装备制造业企业生产技术网络化和系统集成化。改造医药、石化等企业的生产服务流程。鼓励有条件的中小企业拓展电子商务业务。

　　三、做大做强现代服务业,努力扩大有效需求对经济的拉动作用

　　把扩大城乡居民消费作为激活内需的有效途径，促进现代服务业提档升级，提升对经济增长的拉动力。

　　重点发展旅游会展业。实施“双轮驱动，两季繁荣”战略，推动旅游业转型升级，实现由单纯扩张规模向扩大规模与提升效益并重转变。利用承办“大冬会”的契机，推动冰雪旅游基础设施建设，持续扩大冰雪旅游的对外影响力。加快旅游资源的开发利用和区域整合，深度开发森林生态、沿江航游、休闲度假等特色旅游产品。积极落实东北“4+1”旅游联合体《关于建立无障碍旅游区协议》，延伸产业链条，打造精品线路，叫响“冰城夏都”的靓丽品牌。全年接待国内外游客增长19%以上，旅游总收入增长20%以上。高质量办好“哈洽会”、“冰雪节”、“制博会”、“啤酒节”等大型展会和节庆活动，提高会展经济综合效益。

　　优化升级商贸流通业。围绕繁荣城乡消费市场，调整优化商业网点布局，推进区域性中心市场和各类专业批发市场建设，提升商贸业的辐射力和拉动力。推广连锁经营、物流配送等现代流通方式，提高服务水平。落实国家鼓励消费的政策措施，扩大汽车、家电等消费。研究制定支持房地产业发展的政策措施，合理引导住房消费预期，促进供需稳定增长。开发服务性消费，培育新的消费热点。实施“千村万户市场工程”和“家电下乡工程”，积极开拓农村消费市场；培育农村新型流通组织，挖掘农村潜在消费需求，促进农村消费结构升级。适度扩大消费信贷，有效促进预期消费。

　　全力打造大物流平台。围绕构建区域物流中心和对俄国际物流中心，完善物流设施体系和环境体系，支持第三方物流加快发展,引导优势企业将生产制造体系与物流体系剥离。重点推进龙运、香坊铁路集装箱、润恒农副产品等物流园区扩建项目，抓好公路、铁路、航空、航运四大物流港基础设施和物流公共信息平台建设，积极吸引国内外大型物流企业，着力提高物流产业集中度。

　　培育壮大服务外包和中介服务业。推进国家级服务外包基地和平房动漫产业基地建设，完成服务外包核心园区及呼叫中心园区一期工程建设。规范发展会计、律师、公证、投资咨询、资产评估、信用评级、招投标等中介服务业。

　　四、加快科技进步和自主创新，努力打造经济发展新优势

　　坚持把科技进步作为转变发展方式的中心环节和战略基点，强化创新能力建设，加速科技成果向现实生产力转化，推进高新技术产业化。

　　加快高端技术转移转化。整合科技资源，加强多种形式的产学研合作。进一步推进技术研发、科技资源信息共享、科技企业孵化、技术交易、科技投融资5大平台建设，切实提高服务功能和水平。推进国家级生产力促进中心和国际科技合作中心建设。新建市级以上工程技术研究中心3家，新增科技企业孵化器2家，实现技术合同交易额35亿元。

　　支持核心技术和关键技术研发。积极推动企业成为技术创新主体，引导企业把技术创新同结构调整、产业升级、兼并重组结合起来，推动装备制造、电子信息、生物医药、新材料、新能源等重点领域和核心技术科技攻关。以氦气透平高温气冷堆、重型燃气轮机、国产碳纤维材料应用、发电设备、半导体照明材料等关键技术研发为重点，实施100项科技攻关项目。

　　加快高新技术产业发展。加速风力发电、汽车电子、动物疫苗产业自主配套，加快血液制品、抗感染类药物等一批高新技术产品的产业化进程，实现规模发展。继续抓好６大高新技术产业技术联盟，做实做强电站装备、食品安全等一批技术联合体。超前储备一批前沿技术，深挖高新技术产业发展潜力，确保高新技术产业产值增长20%以上，科技进步对经济增长的贡献率达到51%。

　　加强国际科技交流与合作。以对俄科技合作为重点，积极开展国际科技交流与合作，重点抓好特种材料特殊加工关键技术、水陆多用途运输车、强化换热关键技术等一批国家重大科技专项，为哈尔滨市高新技术在国家高端占位奠定基础。

　　营造良好的科技创新环境。加强创新型城市建设目标责任考核，全面落实科技创新政策。创新科技管理体制机制，完善人才激励机制，建设创新人才高地。启动大中型企业知识产权培育工程，提升规模以上企业发明专利拥有量，创建国家知识产权示范城市，实现专利申请量4300件以上。

　　五、继续深化改革扩大开放，努力增强经济发展活力和动力

　　抓住影响和制约发展的关键环节，全力破解体制性障碍，建立完善有利于保增长、促发展的体制机制。

　　突出抓好重点领域改革。进一步深化国有企业改革，全面完成具备条件的厂办大集体企业改制。强化国有资产监管，向12个市属大企业集团派驻监事会，考核国有及国有控股企业负责人经营业绩，建立健全出资人财务监督体系，加强国有资产经营预算管理和收益收缴工作。加快公共交通、公共环境、公共医疗、供热、供气、供水领域改革。

　　支持非公有制经济加快发展。全面落实《关于进一步促进非公有制经济又好又快发展的若干意见》，鼓励、支持和引导非公有制经济进行资源整合、做大做强、加快发展。简化项目审批程序，清理收费项目。在市场准入、投资融资、财税扶持、服务体系等方面给予支持，允许非公资本进入公共事业、基础设施和金融服务等领域，鼓励民营企业参与国有企业战略重组和大项目建设。抓好“中小企业创业成长工程”，将中小企业发展基金由1000万元增加到2000万元，支持中小企业做大做强。创办中小企业5000户，确保非公有制经济实现增加值1625亿元，增长14%以上。

　　努力扩大对外贸易。大力发展加工贸易和服务贸易，扶持重点企业优化出口结构、扩大出口。推动对俄贸易合作提档升级，加快建设对俄出口加工园区，力争再培育5—10户对俄贸易额超千万美元企业。重点扶持具有一定规模实力、品牌优势和市场基础的企业到境外建设加工基地、营销网络和研发机构。在巩固欧、美、俄、日、韩及东南亚等传统市场的同时，积极拓展非洲、拉丁美洲、大洋洲和北美洲等新兴市场。抓住人民币升值的契机，在稳定出口的同时，扩大企业急需设备的进口和先进技术的引进，确保对外贸易总量在外需萎缩的情况下实现稳中有升。

　　大力开展招商引资。把招商引资作为扩大经济增量的主要抓手，继续实施大招商、招大商战略，搞好大项目的专业化包装和国际化运作，提高招商引资成功率。突出招商引资重点，集中推介基础设施、高新技术等重大项目，抢抓发达地区产业结构调整和资本外溢的有利契机，有计划、有选择地承接国际、国内产业转移。完善招商引资机制，建立和落实招商引资奖励办法，调动社会各界参与招商引资的积极性。加大招商项目跟踪服务力度，落实领导包保重点项目责任制，对未落地项目实行责任倒查，确保引资项目不流失。

　　六、加大城乡基础设施建设力度，努力加快宜居城市建设步伐

　　围绕创建国家卫生城、环保模范城、国家园林城和建设宜居城市目标，加大基础设施建设、环境保护和城市环境综合整治力度，打造干净有序文明城市。

　　加强城乡规划管理。以建设“冰城夏都”为目标，开展城市重点问题规划研究，加快城乡一体化发展规划编制，完善松北、群力、哈西新区开发和老城区改造规划。加强呼兰、阿城区控详规划编制，深化细化松花江哈尔滨段“十大工程”近期建设规划。进一步推进保障性住房规划编制工作。加强乡镇、村屯规划编制，促进城乡统筹发展。运用高科技手段进一步加强建设项目联合执法。

　　加快城乡重大基础设施建设。发挥投资对经济增长的直接拉动作用，为工业化、城镇化奠定坚实的物质基础。全力推进地铁一期、松浦大桥等重点工程建设。启动三环西桥、文昌污水处理厂升级改造，持续实施外延性道路打通和城区路网升级达标工程。完成磨盘山供水二期和西泉眼水库维修加固及群力、平房、信义污水处理厂建设。积极配合铁道部推进哈尔滨火车站及周边环境改造和哈尔滨西客站、哈尔滨北客站、哈大客运铁路建设。打好公路建设三年攻坚战，完成绕城高速公路东北环段、鸡讷公路方正至通河段新、改建工程，开工建设哈肇、铁通省级干线公路。建设高等级路面农村公路1500公里、乡镇客运站10个。推进农村饮水安全工程，集中解决13.7万人安全饮水问题。建设沼气工程15处，发展户用沼气池8000个。

　　突出抓好节能减排和生态环境保护重点工程建设。深入推进节能减排。加快推进哈三电厂等12个重点减排工程建设，淘汰10户企业落后产能，加强对20户重点耗能企业节能监察，持续推进既有建筑节能改造工作。加快发展循环经济。重点推进市开发区和利民开发区2个循环经济专业园区建设。着力发展粉煤灰、农作物秸秆、废旧物资再利用等10个循环经济产业链。加强8个重点行业的20家循环经济试点建设，培育18个农业循环经济示范点。加强生态文明建设。全面实行排水许可管理，确保市区和县（市）污水集中收集、集中处理率分别达到100%和80%以上。全面完成松花江规划治污项目，综合整治向松花江直排污水、农药、化肥残留物等污染物的村屯。积极推进国家环保科技产业园建设，重点抓好国家级农村环境保护试点。创建1个省级生态示范区、5个省级环境优美乡镇、18个生态文明示范村、40个环保宜居社区、30个绿色单位和20所绿色学校。

　　千方百计筹集建设资金。充分发挥政府调控的主导作用，加强对沿河沿线土地开发利用的管控，加大城市周边开发建设力度。大力实施老城区腾迁改造。鼓励支持社会力量投资建设路桥、供水和垃圾处理等基础设施，实现城市资源、资产整合配置运营的良性循环。采取建设—经营—移交、资产证券化、委托借款、金融租赁等模式，多渠道、多元化筹集资金，缓解基础设施和公用事业建设资金短缺问题。

　　切实提高城市环境卫生质量。强力推进“干净城市”建设，抓好沿江沿河、城乡接合部、铁路沿线、背街背巷等环境卫生治理。加快推进垃圾无害化处理。实施主干道路大中修、人行道整修工程，安装检查井防护网，提高道路通行能力和安全水平。加快数字城管建设。持续推进行人过街桥、无障碍通道等便民设施建设。植树110万株，新增绿地2100公顷，新增城区集中供热面积600万平方米，全面完成天然气气源转换。推进社区公益用房达标建设。优化公交线网，调整延伸公交线路8条，新增、更新公交车辆150台。实施沿江建筑景观更新改造、夜景亮化等十大工程。整治改造小区庭院500个、社区游园50个，成片推进裸露地软硬覆盖。综合整治闲置废弃锅炉房、烟囱，努力提高城市环境质量。

　　七、推进以改善民生为重点的社会建设，努力促进精神文明建设、保持社会和谐稳定

　　坚持以人为本、民生优先，以惠民行动项目为载体，集中力量办好经济社会发展急需、人民群众热切期盼的实事。

　　全方位促进就业增长。坚持以创业带动就业，加快完善创业服务机制，鼓励和扶持自主创业、自谋职业，发展小企业和创业孵化基地，争创国家级创业型城市。将小额担保贷款总额增至5500万元，范围扩大到所有失业人员，个人小额担保贷款额提高到5万元。完善就业服务体系，强化下岗失业人员再就业和农民工培训。扩大就业援助范围，采取开发公益性岗位等有效措施，安置困难群体就业。建立健全调控预警机制，定期进行失业情况分析预警，搞好失业防控。进一步规范劳动力市场秩序，维护劳动关系双方的合法权益。城镇新增就业8.5万人，转移农村劳动力104万人，确保就业形势基本稳定。

　　加快完善城乡社会保障体系。继续扩大基本养老、失业、医疗、工伤、生育保险的覆盖范围，扩大城镇职工基本医疗保险个人账户支付范围，搞好大学生城镇医疗保险试点，将灵活就业人员纳入失业保险范畴，将农民工、失地农民和农村人口纳入社保体系。强化社会保险基金征缴和监管力度，保证基金安全运行。完善城乡低保制度，建立城乡低保标准动态调整机制，将低收入困难家庭全部纳入救助范围，确保困难群众生活水平不下降。加大社会福利设施建设力度，第一社会福利院新址建成并投入使用，易地新建儿童福利院，建成流浪少年儿童救助保护中心和精神病人托管服务中心。落实“民办公助”政策，鼓励社会力量兴办老年福利机构，新增城镇养老床位3000张，整合建成20所农村敬老服务中心。完善优抚保障制度，提高优抚保障水平。加强防灾减灾救灾工作。切实保障残疾人合法权益。支持慈善事业发展。

　　促进教育均衡协调发展。全面推进素质教育，提高教育教学质量。推进义务教育学校标准化建设，调整学校网点布局，优化资源配置。关心特殊教育，重视学前教育，扶持民办教育，发展高中阶段教育。加强农村寄宿制学校建设。强化职业教育基础能力建设，重点推进轻工业学校及其综合性实习实训基地建设，建设一批示范性、骨干性中职学校。加强师资队伍建设，免费培训5000名农村教师。加大贫困助学工作力度，保障进城务工人员子女享受平等受教育权利。

　　推进医疗卫生事业发展。完善社区卫生机构标准化建设，完成45个社区卫生机构新建、改扩建和71个社区卫生服务机构设备配置。加快农村三级医疗卫生网络建设，全面完成乡镇卫生院房屋改造，免费培训3000名乡村医生。完善新型农村合作医疗制度，投入2.7亿元，筹资标准由人均80元提高到100元，增加慢性病门诊补偿病种，扩大基本药品目录。加强传染病防治，完善公共卫生应急机制，提高应对突发性公共卫生事件能力。建立健全食品药品监管长效机制，全面提高科学监管能力，确保公众饮食用药安全。

　　大力繁荣文化事业。加大公益性文化事业投入，加强博物馆、图书馆、艺术中心等文化基础设施建设，建立10个社区图书馆分馆。广泛开展群众性文化活动，扶持和培育一批大众化、公益性文艺品牌，鼓励文化精品创作。调动社会力量发展文化产业，培育民间工艺文化产品市场，推动旅游文化产业发展。深化文化体制改革，加强历史文化遗产抢救、保护，统筹城乡文化发展。推进有线电视数字化双向整体转换。加强文化市场监管，确保文化市场健康发展。不断解放和发展文化生产力，提升文化软实力和竞争力。

　　改善城乡居民居住条件。改造棚户区100万平方米，新增廉租住房受益户2000户，增加经济适用住房等保障性住房供给。完善和谐拆迁配套政策措施。加快推进农村泥草房改造工程，完成泥草房改造4万户、200万平方米。全年竣工住宅400万平方米。

　　进一步推进维稳创安工作。强化各级政府维护社会稳定安全第一责任。加强“平安哈尔滨”建设和社会治安综合治理，维护国家安全和政治安定。完善城市治安防控体系，深化社区警务和农村治安防范，推进技防工程，提升打击各类违法犯罪活动能力，确保群众生命财产安全。加强安全生产，加大道路交通、消防、矿山、建筑施工、民用爆炸品与危险化学品、特种设备等重点行业领域的隐患排查整治和安全监管力度，强化责任落实和追究，坚决遏制重特大安全事故发生。落实信访责任制，严格执行一票否决制，完善矛盾排查、调解工作机制，拓宽社情民意反馈渠道，引导群众依法表达合理诉求，努力维护社会稳定。加强普法教育和依法治理，努力创造公平正义的法治环境和优质高效的服务环境。

　　高标准、高质量做好“大冬会”承办工作。以基础设施最好、赛事准备最细、各项服务最优、生活环境最佳、观众和市民文明程度最好为目标，加强比赛场馆、驻地宾馆、商业网点、旅游景点、交通设施等重点部位的管理整治，加强城市运行和保障工作，提高场馆运行效率和管理质量，严格排查消防隐患，加强食品安全卫生监管，提高处理疫情和突发事件能力，做好药品、设备应急准备，强化安全保卫工作，确保“大冬会”顺利进行，努力把“大冬会”办成一届成功、完美、大冬会史上最出色的冬季体育盛会。同时，适时做好申办冬奥会准备工作，不断扩大哈尔滨的国际影响力。

　　加大妇女、儿童发展两个《规划》实施力度，推进各项工作终期达标。继续做好人口和计划生育、民族宗教、新闻出版、外事侨务、对台、人防、民兵和预备役、气象、档案、地方志等工作。

　　八、重视和加强财政金融工作，努力发挥财政金融对经济发展的促进作用

　　围绕建设立足黑龙江、面向东北亚的区域性金融中心目标，全力促进金融业发展，努力扩大财政投入规模，为经济社会发展提供资金支持。

　　做实做大融资平台。从整合金融资源、搭建融资平台、吸纳信贷资金、发行企业债券、资本市场融资等方面入手，建立健全为经济建设服务的金融体系。以群力新区开发建设为契机，加快推进“一核一带”金融商务区建设，争取更多国内外金融机构落户哈尔滨市。通过国债、规费注入等形式壮大市投资集团、市城投集团的资金和资产实力，提高融资能力。整合政府资源，积极组建工业、农业和文化等领域的融资平台。指导和扶持条件成熟的区、县（市）建立融资平台，为地方投资建设筹集资金。

　　积极创新融资方式。继续完善创业投资体系，建立产业投资管理体系，全力构建政、银、企“零距离”无缝对接的融资机制，为企业提供高效的融资服务。增强民营企业担保中心担保能力，全面开展工业企业贷款担保风险备付资金业务，进一步扩大信用担保。创新中小企业贷款方式，探索以产权、市场、资产、技术等资源置换资金，争取发行中小企业集合债券。推进江海证券第三步增资扩股，力争有1—2户企业挂牌上市、1—2户企业完成上市申报，鼓励已上市企业再融资。完善社会信用体系，优化金融环境，提高防范金融风险能力。

　　加大财政支持力度。抓住国家扩大内需、加大投资的机遇，积极主动与国家和省对接，争取资金和政策等方面的支持。用足用好财政引导资金，加大财政对农业、结构调整、自主创新、节能减排、中小企业发展、改善民生等方面的投入力度，放大财政资金的带动效应。继续推进项目支出绩效评价、参与式预算、财政国库集中支付等财政管理制度改革，提高财政资金使用效率。高度重视税收征管工作，支持税务机关落实国家各项税收扶持政策，依法履行筹集财政收入、调节经济、调节收入分配职能，切实维护税收政策的统一性和严肃性。

　　九、切实强化政府自身建设，努力提高推动科学发展的能力和水平

　　把实现、维护、发展好人民群众根本利益作为工作的出发点和落脚点，努力在服务中创新管理、在管理中体现服务。保持昂扬奋进的精神状态。以学习实践科学发展观活动为契机，教育广大公务人员解放思想，更新观念，越是在困难的时候，越要坚定不甘人后、赶超先进的信心和勇气。弘扬敢闯敢试、敢于创新的精神，始终保持奋发向上、拼搏进取的工作状态和时不我待、只争朝夕的顽强干劲。全面推行政府绩效考评，对重点工作实行全程跟踪问效，严格落实行政问责制，坚决做到责权统一。

　　提升驾驭经济全局能力。在当前特殊形势下，凸显地方政府调节微观经济职能尤为必要。要深刻把握社会主义市场经济的内在规律，寻找经济运行中政府有形之手与市场无形之手的最佳结合点，双管齐下，优势互补，激活新一轮经济增长周期的驱动引擎。在全面落实国家和省出台的保增长、促发展、调结构政策措施的同时，针对企业遇到的实际困难，逐企业、逐项目、逐问题地研究制定帮扶政策，发挥政府在化解危机、防范风险、推动发展中的作用，政企合力应对挑战、渡过难关。

　　努力提高行政效能。做好大部制改革的准备工作，科学谋划、适时启动机构改革，理顺部门职能，推动职能转变。坚持依法行政，实行行政处罚自由裁量权基准制度，做好相对集中行政复议权试点工作。强化对政府工作运行的监督，坚决查办决策执行不作为、乱作为、“中梗阻”和冷、横、硬问题。大幅压缩会议讲话、政府文件、临时机构、公费出国和公务消费，确保行政办公经费“零增长”。继续减少审批项目、前置要件和审批环节，有效解决行政审批“体外循环”问题，推行限时并联审批和超时默许制，构建名副其实的“一站式”审批服务体系。

　　切实加强廉政建设。加大反腐倡廉力度，严格落实党风廉政建设责任制。在坚决惩治腐败的同时，更加注重治本，更加注重预防，加大对失职渎职行为的责任追究力度，加强对专项资金、重大投资项目和权力部门的审计和监察，坚决查处官商勾结、权钱交易等违纪违法案件，推进廉洁高效政府建设，为改革发展稳定提供坚强保证。

　　各位代表，我们处在一个新的历史起点上，面对前所未有的严峻挑战，我们一定要更加紧密地团结在以胡锦涛同志为总书记的党中央周围，更加坚定自觉地深入贯彻落实科学发展观，紧紧依靠全市人民，同心同德，共度时艰，变压力为动力，化危机为机遇，奋力推进经济社会又好又快发展，努力建设“三个适宜”现代文明城市，以优异的成绩向建国60周年献礼！
